# Resistance to Neuromuscular Blockade by Rocuronium in Surgical Patients with Spastic Cerebral Palsy

**DOI:** 10.3390/jpm11080765

**Published:** 2021-08-03

**Authors:** Stephanie Lee, Karyn Robinson, Madison Lodge, Mary Theroux, Freeman Miller, Robert Akins

**Affiliations:** 1 Nemours Biomedical Research, Nemours-Alfred I. duPont Hospital for Children, Wilmington, DE 19803, USA; stephanie.lee@nemours.org (S.L.); karyn.robinson@nemours.org (K.R.); mlodge@usc.edu (M.L.); 2 Department of Anesthesiology, Nemours-Alfred I. duPont Hospital for Children, Wilmington, DE 19803, USA; mary.theroux@nemours.org; 3 Department of Orthopedics, Nemours-Alfred I. duPont Hospital for Children, Wilmington, DE 19803, USA; Freeman.Miller@nemours.org

**Keywords:** drug resistance, cerebral palsy, rocuronium, skeletal muscle

## Abstract

Individuals with spastic cerebral palsy (CP) often exhibit altered sensitivities to neuromuscular blocking agents (NMBAs) used for surgical intubation. We assessed usage of the NMBA rocuronium in patients with spastic CP and evaluated potential modifiers of dosing including gross motor function classification system (GMFCS) level, birthweight, gestational age, and the use of anticonvulsant therapy. In a case-control study, surgical patients with spastic CP (*n* = 64) or with idiopathic or non-neuromuscular conditions (*n* = 73) were enrolled after informed consent/assent. Patient data, GMFCS level, anticonvulsant use, and rocuronium dosing for intubation and post-intubation neuromuscular blockade were obtained from medical records. Findings reveal participants with CP required more rocuronium per body weight for intubation than controls (1.00 ± 0.08 versus 0.64 ± 0.03 mg/kg; *p* < 0.0001). Dosing increased with GMFCS level (Spearman’s rho = 0.323; *p* = 0.005), and participants with moderate to severe disability (GMFCS III-V) had elevated rocuronium with (1.21 ± 0.13 mg/kg) or without (0.86 ± 0.09 mg/kg) concurrent anticonvulsant therapy. Children born full-term or with birthweight >2.5 kg in the CP cohort required more rocuronium than preterm and low birthweight counterparts. Individuals with CP exhibited highly varied and significant resistance to neuromuscular blockade with rocuronium that was related to GMFCS and gestational age and weight at birth.

## 1. Introduction

Cerebral Palsy (CP—see table of abbreviations below) is a group of disorders occurring in approximately 2.9 per 1000 children [1,2]. CP is characterized by a static, non-degenerative encephalopathy arising in the developing brain with associated musculoskeletal and peripheral effects that often worsen over time [1,3,4,5]. Personalized care for children with CP is challenging due in large part to the complex, multifactorial nature of the condition and a lack of understanding of the fundamental pathways contributing to disease. A number of risk factors for CP have been identified, including prematurity, hypoxia-ischemia, placental insufficiency, chorio-amnionitis or other prenatal infection, perinatal inflammation, genetic causes, and combinations of these factors [6,7,8,9]. In particular, the presence of genetic variants have been investigated in some types of CP [10,11] with de novo copy number variants [12,13,14,15,16,17], single nucleotide variants [18], and de novo mutations [19] implicated in as many as 33% of the CP cases evaluated in those studies. Additional studies suggest that epigenetic alterations are associated with CP [20,21,22,23]. Improvements in personalized care based on these advances will require more complete delineation of the characteristics and susceptibilities among individuals with different types of CP. 

Children with spastic CP, which accounts for about 77% of all cases, often require multiple surgeries throughout their lifetimes due to progressive motor and musculoskeletal dysfunction [1,2,24,25,26]. Surgical correction of gait abnormalities, relief of muscle hypertonia, bone osteotomies, device implants, and posterior spinal fusions are common for individuals with CP [27,28]. Neuromuscular blockade is required for intubation and, in some cases, to maintain muscle relaxation during surgery. Non-depolarizing neuromuscular blocking agent (NMBAs), which inhibit nicotinic acetylcholine receptor (nAChR) at neuromuscular junctions (NMJs) [29], are often used. Rocuronium bromide (ROC), is a preferred NMBA because of its rapid onset and intermediate duration of action [30]. Standardized doses of ROC (0.45 to 0.6 mg/kg of body weight) typically achieve the neuromuscular blockade needed to facilitate intubation [31].Studies show that patients with CP exhibit altered sensitivities to NMBAs [32,33,34,35,36]; however, ROC dosage variability in individuals with CP is not well understood [33,37]. 

The relationship between individualized dosing with ROC, which is generally determined in the operating room in real time based on measurement of neuromotor blockade and titrated up if needed, and the degree of motor involvement in children with spastic CP has not been investigated. Anticonvulsant therapy, which is common in CP due to co-occurring seizure disorders [1,38] has demonstrated an association with altered responsiveness to NMBAs such that some patients on chronic anticonvulsant therapy (CAT) recovered significantly faster from the same single dose of ROC as their control counterparts despite achieving the same level of initial neuromuscular blockade [39]. A separate study comparing effects of continuous dosing of ROC between patients on CAT and controls, revealed that patients on CAT required higher infusion doses to maintain neuromuscular block resulting in zero twitch response [40]. Sheshadri et. al. added that the elevated infusion requirements in patients on CAT were independent of serum levels of phenytoin and while not significant, also reported that recovery from neuromuscular block was faster in the CAT group as compared to controls [40]. Additional studies have found that utilization of ROC in patients on CAT may be due in part to long term effects of CAT on liver hypertrophy and higher levels of cytochrome P450 (CYP) [41], and CYP3A4 was found to interact with ROC [42], suggesting that the elevated need for ROC in patients on CAT may be associated with elevated liver enzymes that sequester ROC. While it has been shown that CAT can affect NMBA dosing requirements, the variability among patients is not well understood, and it remains unclear why patients with CP who are not on CAT might require more ROC for intubation than controls [32,39]. The present study was designed to elucidate associations between CP severity, anticonvulsant use, and ROC dosing in surgical patients with spastic CP and to evaluate potential contributions of CP-associated risk factors and characteristics to altered ROC dosing.

## 2. Materials and Methods

### 2.1. Participants and Data

**Figure 1:**
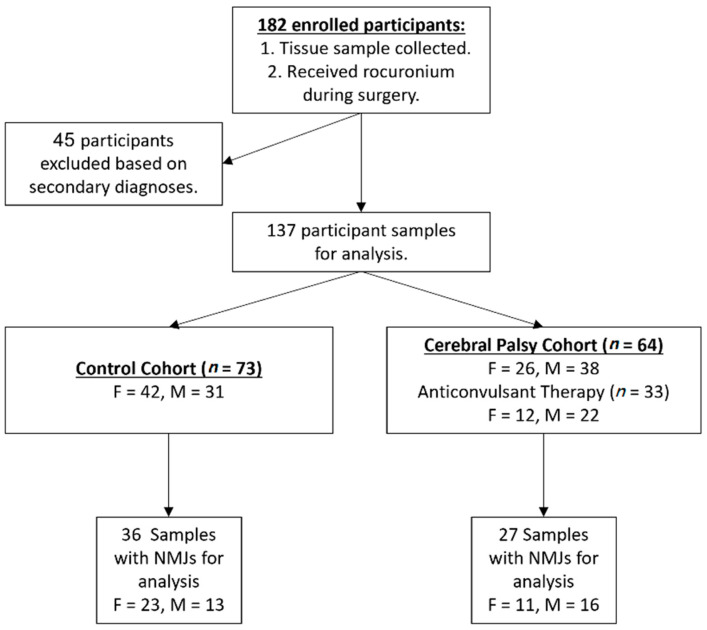
Participant enrollment. Of the original 182 enrolled participants, a total of 137 patients were included in the analyses after 45 patients were excluded due to the appearance of exclusionary secondary diagnoses, missing data in the electronic medical record (EMR), or a change in primary diagnosis. Data analysis included *n* = 73 controls and *n* = 64 participants with CP. The number of female (F) and male (M) participants included in each cohort are indicated. All enrolled patients were utilized in the ROC dose analysis and for analyses comparing ROC dose against gestational data. Muscle sections from each diagnostic group were scanned for the presence of at least 10 nAChRs per section. Samples with fewer than 10 nAChRs per section were excluded from NMJ analysis.

Patients scheduled for spine surgery at the Alfred I. duPont Hospital for Children in Wilmington, DE, between 2013 and 2017 and having a diagnosis of spastic di- or quadriplegic CP (cases) or a non-neurodegenerative condition associated with either juvenile or adolescent idiopathic scoliosis or pars interarticularis defect (controls) were enrolled after consent and assent under the following IRB approved studies: The Pathophysiology of Physical Disability in Cerebral Palsy (IRB#687629), The Neuro-Orthopedic Tissue Repository (IRB#381494), and The Nemours Biobank (IRB#349465). 182 participants receiving ROC during surgery were enrolled; 45 participants were subsequently excluded due to missing medical record data or the presence of a secondary diagnosis of neuromuscular scoliosis or a chromosomal or genetic abnormality (Figure 1). Patients with a diagnosis of CP were separated into two sub-groups based on whether they were receiving anticonvulsant therapy at the time of enrollment/surgery or not. Pre-operative levels of liver function markers (alanine transaminase (ALT) and bilirubin) were noted due to their association with resistance to neuromuscular blockade induced by chronic anticonvulsant therapy [39,41,42]. Diagnostic, demographic, and other descriptive data were collected from the EMR.

### 2.2. Tissue Preparation, Staining, and Imaging

Muscle samples were obtained during surgery using a surgical scalpel. Samples from erector spinae were taken from the concave side of the scoliotic curve at the thoraco-lumbar junction. Lower extremity samples were obtained from muscle tissue that was adjacent to the tendon being modified or resected during the procedure. samples were collected by the lead surgeon during the procedure and placed in a sterile specimen cup unless the surgeon decided that the progression of the case, the local anatomy, the status of the patient, or other considerations disallowed the collection. Tissue was transferred to the lab, trimmed and mounted using tragacanth to allow the preparation of ≤1 cm^2^ cross-sections, cryopreserved by immersion in liquid nitrogen chilled isopentane, and stored at −80 °C. Sections were prepared by the Nemours Histotechnology Core for immunostaining; additional 8–10 µm sections were post fixed in 10% neutral buffered formalin and stained with hematoxylin and eosin to assure tissue integrity, the presence of cross-sectioned muscle, and the absence of freezing artifact.

Samples were evaluated using 3 NMJ markers: nicotinic acetylcholine receptor (nAChR), acetylcholinesterase (AChE), and laminin β2 (Lβ2) with primary (monoclonal anti-Lβ2) and secondary (goat-anti-mouse IgG with Alexa 488) antibodies along with fluorescent probes for nAChR (α-bungarotoxin) and AChE (fasciculin-2) [43]. Slides were fixed for five minutes with 2% paraformaldehyde (Electron Microscopy Sciences) in Dulbecco’s Phosphate-Buffered Saline (DPBS, pH 7.0–7.6, Thermo Fisher Scientific). All subsequent reagents were prepared in DPBS with DPBS rinses performed between steps. The tissue samples were permeabilized with 0.1% Triton X-100 (Sigma-Aldrich) for 15 min, and blocked with 3% bovine serum albumin (Sigma-Aldrich) for 30 min at room temperature. After tissue sections were fixed and permeabilized they underwent screening for the presence of at least 10 NMJs (Control = 36 and Cerebral palsy = 26) using fluorescently-tagged α-bungarotoxin. Sections containing 10 or more nAChRs (as labeled by α-bungarotoxin) were stained for one hour at room temperature with a monoclonal primary antibody to locate Lβ2 using a solution containing 4 mg/mL C4 supernatant prepared per instructions from the Developmental Studies Hybridoma Bank (DSHB) in Iowa City, Iowa (The hybridoma, C4 developed by J.R. Sanes, was obtained from the Developmental Studies Hybridoma Bank created by the NICHD of the NIH and maintained at The University of Iowa, Department of Biology, Iowa City, IA 52242). Sections were rinsed and incubated with a secondary antibody (Alexa Fluor 488-conjugated goat-anti-mouse IgG at 1:500 dilution, Life Technologies) and directly labeled peptides to illuminate acetylcholinesterase (AChE; Fasiculin-2, Abcam, at 1 µg/mL, directly labeled with Alexa Fluor 555 Microscale Protein Labeling Kit, Life Technologies) and nicotinic acetylcholine receptor (AChR; Alexa Fluor 647 conjugated a-Bungarotoxin at 0.16 µg/mL, Life Technologies). Slides were mounted with anti-fade solution (Life Technologies) and sealed under #1.5 glass coverslips (Fisher Scientific).

Images were acquired on a wide-field Olympus BX-60 fluorescence microscope fitted with a 12-bit Evolution QEi camera (Media Cybernetics). Optical sections were captured using a 40× PlanApo objective and a computer-controlled stage using Image Pro Plus Software 6.3 (Media Cybernetics). The co-mapping of marker pairs was determined as in previous studies [43,44]. Composite images of nAChR with AChE, nAChR with Lβ2, and AChE with Lβ2 were pseudo-colored red and green (respectively per pairing) such that colocalized areas were yellow. Pairwise NMJ organization scores were calculated as the proportion of voxels containing only one marker (red or green) to the total number of voxels with that stain (red + yellow or green + yellow) to estimate the degree of NMJ disorganization (a score of 1 indicated complete separation of the two components; a score of zero indicated complete co-localization).

### 2.3. Statistics

GraphPad Prism (Version 6.07) was used for statistical analysis. For continuous variables, parametric testing included two-way and one-way analysis of variance (ANOVA) with Holm-Sidak’s multiple comparison test. Nonparametric testing included Mann Whitney U (MWU) and Kruskal Wallis (KW) testing. For correlations, Pearson’s r was used to evaluate two continuous variables; otherwise, Spearman’s rho was used. Fisher’s Exact Test (FET) was employed to test contingencies. Statistical *p*-values below 0.05 were considered significant. The test applied is indicated for each comparison. Unless otherwise noted, data are provided as means with standard error of the mean (SEM) or medians with range. Cases with missing data were excluded from analyses.

## 3. Results

### 3.1. Demographics

Of the 182 participants enrolled, 45 were excluded due to missing data or potentially confounding secondary diagnoses. Demographic data on the remaining 137 participants are summarized in Table 1. While there were significant differences in age and body mass index (BMI) between the case and control cohorts (*p* < 0.001 for both by MWU test), these characteristics did not correlate with the dose of ROC for either group (CP: age, *p* = 0.412 and BMI, *p* = 0.256; Control: age *p* = 0.220 and BMI *p* = 0.634, Spearman’s rho). In addition, the distribution of females and males in the groups was not significantly different (*p* = 0.06, FET); the participants’ sex within the cohorts was not significantly related to ROC dosing and did not have a significant interaction with diagnosis (*p* = 0.172 and *p* = 0.287, respectively, by 2-Way ANOVA).

### 3.2. Rocuronium Dose Determination

The doses of ROC utilized for intubation and for post-intubation maintenance of neuromuscular blockade were derived from electronic surgical records. Clinically, NMBA dosing is often titrated based on an individual’s response to induced muscle contraction until optimum neuromuscular blockade is reached [45,46]. For participants in the present study, ROC dosages were determined in this way at the time of the procedure using a standard practice where an initial dose was calculated based on body weight and additional ROC added by titration if needed. In short, well-established methods of train-of-four ulnar nerve stimulation to induce contraction of the adductor pollicis were used [36,47]. This process allowed sufficient relaxation while avoiding the use of excessive ROC, which can be problematic in post-surgical recovery [48,49]. The primary use of ROC was to facilitate intubation, and ROC was generally discontinued thereafter to allow neurophysiologic monitoring of the spinal surgery. In cases where neurophysiologic monitoring was not being performed or was not possible, intraoperative doses of ROC were sometimes delivered to help maintain optimal surgical conditions and reduce involuntary patient movements, spontaneous respiration, or spasmodic breathing [31,50].

A total of 25 different anesthesiologists, with approximately equal CP and control cases, were responsible for administration of the intubating rocuronium dose. We found no significant effects on ROC dosing evaluating anesthesiologist alone or when comparing the effect of anesthesiologist on the ROC dose of patients categorized by GMFCS (data not shown: TWA, *p* = 0.24 and *p* = 0.27 respectively). Subsequent sensitivity analysis of total anesthesiologists was performed in which the correlation of mean ROC dose per GMFCS against mean ROC dose per GMFCS with groups of 5 anesthesiologists removed. This sensitivity analysis revealed no effect of anesthesiologist on rocuronium dose as each correlation yielded a significant Pearson correlation coefficient (Supplemental Figure S1).

### 3.3. Rocuronium Dose and Degree of Neuromotor Impairment

Overall, individuals with CP required approximately 1.6 fold more ROC for intubation than controls (Figure 1; cases =1.00 ± 0.08 and controls = 0.64 ± 0.03 mg/kg; *p* < 0.0001, MWU). Among patients with CP, GMFCS scores, which indicate the degree of neuromotor impairment [51], correlated significantly with intubating ROC dose (Figure 1, Spearman’s rho = 0.3423 *p* = 0.005). In 32 CP and 7 control surgeries, maintenance doses of ROC were delivered after intubation. The amount of additional ROC administered was much higher in the CP patients (CP = 1.29 ± 0.18 versus controls = 0.15 ± 0.02 mg/kg, *p* < 0.0001, MWU).

### 3.4. Chronic Anticonvulsant Therapy

Chronic anticonvulsant therapy (CAT) is common among individuals with spastic CP due to a co-occurring seizure disorder or epilepsy [38,52,53,54,55]. Resistance to neuromuscular blocking agents has been linked to the hepatoxicity of anticonvulsants and the upregulation of drug-metabolizing liver enzymes, especially CYP3A4, which is known to metabolize ROC [39,41,42], Although it was not possible to biopsy liver samples for the present study, elevated CYP3A4 generally correlates with increased circulating alanine transaminase (ALT) and total bilirubin [42,56]. Pre-operative serum ALT and bilirubin values were available from the EMR of a subset of study participants receiving CAT (*n* = 29 CP cases) and were evaluated as a surrogate measure against participants who were not on CAT (*n* = 14 CP cases). For patients on CAT, bilirubin and ALT correlated with each other (Spearman’s rho = 0.599, *p* < 0.0003), but there was no significant correlation between intubating ROC dose and serum values of either ALT or bilirubin for any group. Interestingly, CP patients on CAT actually had significantly lower total serum bilirubin than those not on CAT (0.27 ± 0.03 vs. 0.54 ± 0.08 mg/dL, respectively, MWU *p* = 0.0006, Table 2). In addition, although different anticonvulsants have varying degrees of hepatotoxicity risk [57], no significant differences in ROC dose were observed when patient anticonvulsant use was categorized by hepatotoxicity classification (3 classification groups, KW, *p* = 0.718, Supplemental Table S1C).

**Figure 2:**
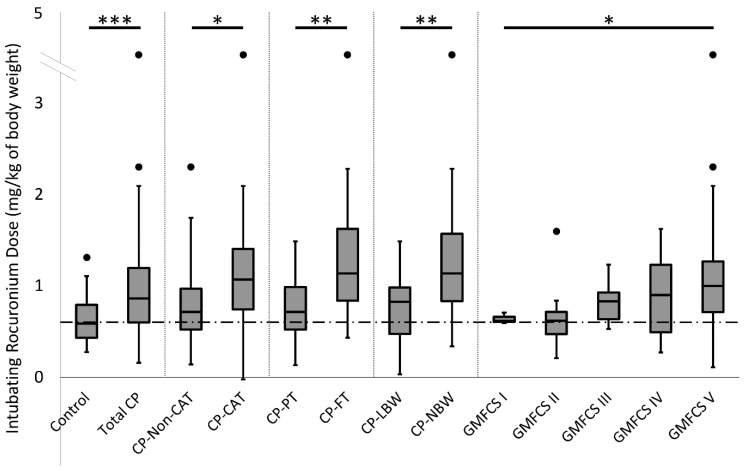
Intubating rocuronium dosing. Box and whiskers plots representing min and max and 1st, 2nd, and 3rd quartiles are shown. Values that were more than 2 standard deviations away from the mean are shown as separate points. Intubating ROC doses were evaluated among study participants in different groupings, which are separated by vertical dashed lines. These include all controls (Control *n* = 73) and all CP (Total CP *n* = 64), participants with CP who were or were not on anticonvulsant therapy (CP-Non-CAT, *n* = 31 and CP-CAT, *n* = 33), children with CP born preterm or full term (CP-PT, *n* = 37 and CP-FT, *n* = 25), children with CP born with low birthweight or normal birthweight (CP-LBW, *n* = 28 and CP-NBW, *n* = 24), and children with CP across the spectrum of GMFCS scores (*n* = 3, 6, 6, 14, and 35 for GMFCS I, II, III, IV, and V, respectively). CP participants required significantly more ROC than controls (*p* < 0.0001, MWU) and well above the standard 0.6 mg/kg dose (represented by horizontal dashed bar). Within the CP cohort, patients who were on chronic anticonvulsant therapy (CAT) required significantly more ROC than those who were not using anticonvulsants (*p* = 0.007, 2WA). Participants with CP who were full-term or were normal birthweight required significantly more ROC than pre-term or low birthweight (*p* = 0.0017 and *p* = 0.0011, respectively, MWU), and ROC dose was significantly correlated with increasing GMFCS score (*p* = 0.005; Spearman’s rho). Filled circles represent outliers. Statistical significance is denoted by: *p* < 0.05 = *, *p* < 0.01 = **, and *p* < 0.001 = ***.

We also found that participants with moderate to severe CP (GMFCS III, IV, or V) had elevated intubating ROC doses whether they were receiving CAT (1.21 ± 0.13 mg/kg; *n* = 30) or not (0.86 ± 0.09 mg/kg; *n* = 19). Both groups differed significantly from each other and from controls (0.64 ± 0.03 mg/kg; *n* = 73) by ANOVA with post hoc Holm-Sidak test: CP-CAT vs. CP-Non-CAT, *p* = 0.03; CP-CAT vs. Con, *p* < 0.0001; CP-Non-CAT vs. Con, *p* = 0.04. Importantly, participants with CP who were not on CAT still required significantly more ROC than controls (Figure 2 and Supplemental Table S1A,B).

### 3.5. Risk Factors Associated with Cerebral Palsy

In many cases, the cause of CP is unknown, but for 46 of our participants, a suspected etiology was indicated in their EMR (Table 1). Among these 46, no significant associations between GMFCS score and suspected etiology were observed (data not shown), and no effect of etiology was seen on ROC dose (Supplemental Table S2).

Birth at an early gestational age (GA) and low birthweight (BW) are risk factors for CP [58]. Accordingly, ROC doses were analyzed for CP participants who were preterm (GA < 37 wks) or low birthweight (LBW; BW < 2.5 kg). Participants with CP born full-term required significantly more ROC for intubation than those born pre-term (1.29 + 0.16, *n* = 25 versus 0.79 + 0.06 mg/kg, *n* = 37; *p* = 0.0017, MWU). More ROC was also required in CP cases with normal birthweight (NBW) than those with LBW (1.31 + 0.17 mg/kg versus 0.76 + 0.06 mg/kg, *p* = 0.0011, MWU). Interestingly, among subjects with CP who were not on CAT, intubating ROC dose and total ROC dose correlated well with GA (Pearson’s r = 0.416, *p* = 0.022; and r = 0.432, *p* = 0.017, respectively) and BW (r = 0.544, *p* = 0.006; r = 0.586, *p* = 0.003, respectively), but these measures did not correlate well for children taking anticonvulsants or for the control cohort (Table 1 and Table 2 and Supplemental Figure S2).

### 3.6. Rocuronium Dose and Neuromuscular Junction Microanatomy

**Figure 3:**
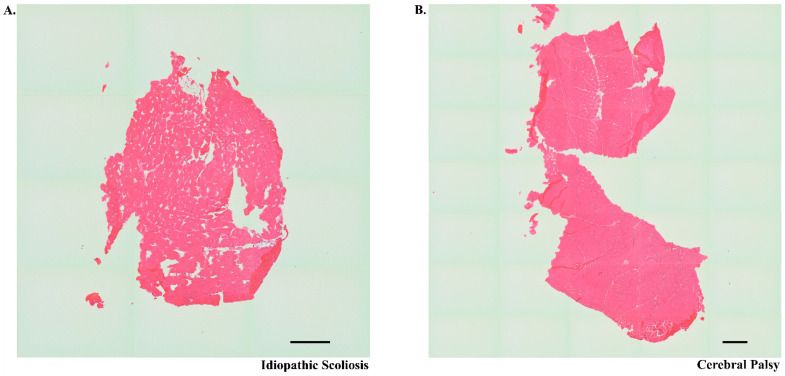
Representative hematoxylin and eosin (H&E) stained muscle tissue. H&Es from a patient with idiopathic scoliosis (panel (**A**)) and cerebral palsy (panel (**B**)) correspond to tissues from which images represented in Figure 4 were obtained. Whole tiled images were captured at 10× magnification on an Evos microscope (scale bar in each photo represents 500 µM.) These images show the general morphology of the muscle samples used in the study.

**Figure 4:**
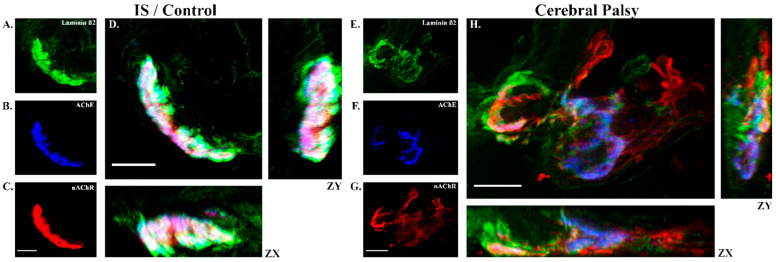
Representative neuromuscular junctions from control and cerebral palsy. Skeletal muscle cryosections were immunofluorescently labeled to illuminate the co-localization of laminin β2 (green; panels (**A**,**E**)), AChE (blue; panels (**B**,**F**)), and nAChR (red; panels (**C**,**G**)). Co-staining displays as white or lavender tones (**D**,**H**) when the color channels are highly co-localized. On average, NMJs from participants with CP (e.g., right panel) exhibited significantly disrupted NMJ organization as compared to controls (left panel; *n* = 200 total NMJs, overall). Disorganization among CP NMJs was evident in the ZY-ZX planes as well. Scale bar represents 10 µM.

Non-depolarizing NMBAs like ROC competitively inhibit nicotinic acetylcholine receptors (nAChRs) organized at NMJs on skeletal muscle [37], Histological screening of muscle samples by hematoxylin and eosin staining was performed to verify that samples were primarily muscle without significant freezing artifact, physical damage from processing, or tissue necrosis (Figure 3). Analysis of immunofluorescence results showed expected findings of dysmorphic NMJs in CP muscle samples such as an increased staining of laminin and disorganization of nAChRs [37,43,44]. To evaluate nAChR distribution and NMJ organization, nAChR was stained and analyzed against other NMJ components acetylcholinesterase (AChE), which is a tightly-regulated enzyme that halts neurotransmission by cleaving acetylcholine [59], and laminin β2 (Lβ2), which is a component of the muscle basal lamina associated with NMJ structure (Figure 4) [60].

**Figure 5:**
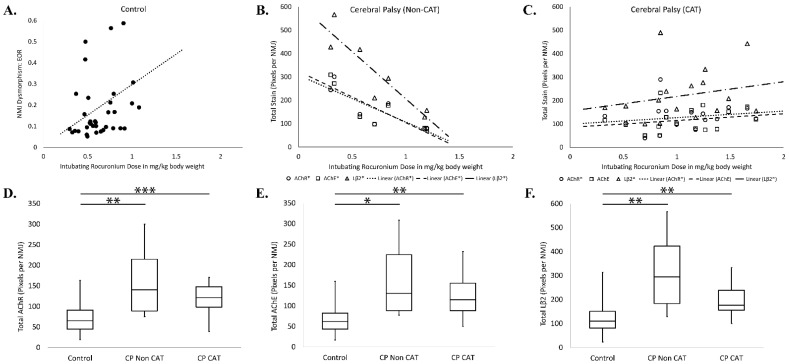
NMJ dysmorphism and total stain versus ROC utilization. NMJ dysmorphism scores and total stain distribution for each NMJ component (E = Acetylcholine Esterase; L = Laminin β2; R = Acetylcholine Receptor) were correlated against the intubating rocuronium dose. Statistical significance among the correlations and distribution comparisons are denoted by stars (*, **, and ***, representing values of *p* < 0.05, 0.01, and 0.001 respectively). Dysmorphism scores were quantified as the proportion of voxels for one stain (outside of another. For example, EOR = proportion of voxels stained for AChE alone to the total of AChE positive voxels stained for AChE alone plus those co-stained for AChE and nAChR. Intubating rocuronium doses for participants with controls significantly correlated with the finding of AChE outside of nAChR ((**A**), Pearson r = 0.338, *p* = 0.022). Intubating rocuronium dose for patients with CP who were not on CAT negatively correlated with total stain distribution for R, E, and L ((**B**), Pearson r= −0.840, −0.851 and −0.908, *p* = 0.009, 0.008, and 0.002, respectively). Intubating rocuronium dose for patients with CP who were on CAT correlated with total stain distribution for R and L ((**C**), Pearson r = 0.394 and 0.428, *p* = 0.034, and 0.030 respectively.) Plots of the relative volume of each NMJ stain (i.e., number of voxels normalized to patient body weight) show both sub-groups with CP significantly differ from controls ((**D**), AChR, CP-CAT vs. Controls, *p* < 0.000, CP-Non CAT vs. Controls, *p* = 0.006; (**E**), AChE, CP-CAT vs. Controls, *p* = 0.002, CP-Non CAT vs. Controls, *p* = 0.025; (**F**), Lβ2 CP-CAT vs. Controls, *p* < 0.001, CP-Non CAT vs. Controls, *p* = 0.003).

Patients with CP whether they were on CAT or not, were found to have statistically larger areas of AChR, AChE, and Lβ2 per NMJ than controls (CP-Cat vs. Control, *p* < 0.000, *p* = 0.005, and *p* = 0.001; CP-Non Cat vs. Control, *p* = 0.006, *p* = 0.025, *p* = 0.003, respectively); however, the CP-subgroups were not significantly different from each other (CP-CAT vs. CP-Non CAT *p* = 1 for all measures, Figure 5D,F). Interestingly, patients with CP exhibited significant correlations between intubating ROC dose and measures of NMJ size as estimated by the extent of AChR, AChE, and/or Lβ2 staining. Both the CP-CAT cohort (Pearson r = 0.394/*p* = 0.043 for AChR; r = 0.226/*p* = 0.428 for AChE; r = 0.169/*p* = 0.030 for Lβ2) and the CP-Non CAT cohort (r = –0.840/0.009 for AChR; –0.851/ 0.008 for AChE; –0.908/0.002 for Lβ2) exhibited correlations between ROC and NMJ size (Figure 5B,C and Supplemental Figure S3B). Among controls, on the other hand, there was no significant correlation in these measures (Pearson r = 0.077/*p* = 0.328 for AChR; r = 0.177/*p* = 0.150 for AChE; r = 0.129/*p* = 0.226 for Lβ2; Supplemental Figure S3A). Thus, ROC dosing seemed positively correlated with NMJ size in the cohort of CP patients on CAT but negatively correlated with NMJ size among CP cases that were not on CAT.

The degree of pairwise co-localization between nAChR, AChE, and Lβ2 was also analyzed for correlation with ROC dose. Not surprisingly, control subjects exhibited a correlation between intubating ROC dose and NMJ dysmorphism represented by the presence of AChE outside of the nAChR-defined NMJ (Pearson r = 0.338, *p* = 0.022, Figure 5A), and there appears to be variability in NMJ colocalization scores among individuals that may account for the relatively small range of doses seen in the control cohort. There were no correlations between intubating ROC dose and NMJ dysmorphism for either CP subgroup. (Supplemental Table S3). Quantification of NMJ dysmorphism showed significant variability (Supplemental Tables S3 and S4), but participants with CP generally showed disrupted NMJ staining patterns compared to controls (Figure 4 and Supplemental Video S1).

## 4. Discussion

Our study expands on previous reports of general resistance to NMBAs in CP through detailed characterization of resistance to ROC in spastic CP [32,35,37]. Participants with CP exhibited elevated ROC requirements and increased variability in ROC dosing associated with GMFCS severity and select birth characteristics. Because higher GMFCS score may be indicative of worsening muscle pathology, we hypothesized that elevated ROC dose in participants with spastic CP, especially those not on CAT, would be associated with NMJ disruption. Our results showed that measures of microanatomic NMJ dysmorphism were associated with ROC dosing; however, the associations varied between the two primary CP-subgroups—those receiving CAT and those not. The CP cohorts also exhibited significant differences in the area of NMJ component staining (AChR, AChE, and Lβ2) that was not see in controls. Together, these data indicate that individuals with CP (whether on CAT or not) may possess disrupted NMJs as has been suggested by previous studies [37,43,44]. More detailed assessments of NMJ component organization and NMJ function may be needed in the future to determine if NMJ disruption is causally related to an increase in the need for ROC in CP.

The use of anticonvulsant therapy further elevated ROC dosing when compared against controls, but patients with moderate to severe spastic CP required significantly more rocuronium for intubation (1.6×) whether they were on CAT (1.9×) or not (1.3×). Interestingly, we expected that participants with CP who were on CAT would show a correlation between elevated ROC dosing and serum ALT, which is a surrogate indicator of the liver dysfunction that has been linked increased ROC catabolism by CYP3A [39,41,42]. This expectation was bolstered by previous reports suggesting that individuals on CAT should exhibit a quicker recovery from neuromuscular blockade whether they received bolus dosing or continuous infusion [39,40]. We found that this expectation was only partly true for participants on CAT (Table 2) as intubating ROC doses did not correlate with ALT; whereas, in cases where ROC was used for extended periods during a case, the total ROC correlated with serum ALT. Regardless of CAT status, participants with CP in our study received significantly more ROC whether it was used for intubation alone or it was used throughout their procedure, as compared to controls. These findings expand previous reports (e.g., [33]) indicating that patients with CP have altered sensitivities to NMBAs and suggest that patients with CP, independent of CAT, require elevated intubating and maintenance doses to provide optimum surgical conditions.

ROC doses in CP correlated with GMFCS scores and with the participant’s gestational age and weight at birth (Figure 2). Elevated total ROC appeared to be independent of surgery duration (Table 1) and unrelated to the relative quantity of nAChR present at NMJs (Figure 5). We also noted an incidental finding that serum levels of magnesium and triglycerides, (Table 2), which have been implicated in ROC dosing, did not correspond with elevated ROC use. In fact, the high-normal serum magnesium and low serum triglycerides seen in CP participants both suggest a lower requirement for ROC [61,62]. As expected, CAT had a significant effect on ROC dosing (Figure 2), but participants with CP who were not on anticonvulsants also had significant resistance to ROC. The non-CAT group showed significant positive correlations between ROC and GMFCS, birthweight, and gestational age at birth. Thus, resistance to ROC in CP appears to be a complex result of multiple inputs, including the use chronic anticonvulsant medication, but children with spastic CP appear also to have a fundamental resistance associated with their diagnosis. Importantly, ROC dosing in the CP cohort also had a high degree of variability, especially as disease severity (i.e., GMFCS score) increased. The combination of increased resistance to ROC and high variability may have implications when administering care to individuals with spastic CP, and clinicians should be prepared for inconsistent patient responses to standardized doses of ROC, and perhaps other drugs, that may vary to a great degree across individual patients.

Interestingly, although low BW and prematurity are risk factors for spastic CP [58], we found that children with CP who were full-term or who had normal birthweights tended to require higher ROC doses during surgery later in life than their preterm and LBW peers. The increased need for ROC in these cases may reflect differences in the etiologies of CP that prevail in late gestation and/or perinatal onset. Studies suggest that term-born individuals with CP are significantly more likely to have birth defects, perinatal stroke, poor fetal growth [63], placental disorders [64], and maternal or neonatal infection [65], while those with CP born pre-term are more likely to have intraventricular hemorrhage or periventricular white matter injury [66]. Although we did not see significant correlations between disease etiology and GA, BW, or total ROC dose in our cohorts, our analyses may be limited by the small number of participants for which an etiology was indicated in the EMR (*n* = 36) as well as an overrepresentation of severely affected participants (e.g., 76.6% of CP participants in our study were GMFCS IV or V compared to 27–31% of such patients that would be expected based on a published meta-analysis of prior CP studies) [67]. Nonetheless, the notion that CP is clinically different in children born preterm versus full-term is consistent with previous studies indicating that severe intellectual disability, epilepsy, and severe ambulatory impairments are more common in term-born than preterm children with CP [68,69].

The positive correlation between GMFCS score and ROC dose (Figure 2) in the CP cohort indicates to us that higher levels of disability in spastic CP are associated with increasing ROC requirements and suggests that functional status and drug resistance are related. Previous data indicate that NMJ dysmorphism is also related to GMFCS [44], and since ROC binds nAChR at NMJs [70], we hypothesized that NMJ disruption would be related to ROC dosing. Although we found some correlation between ROC dosing and total NMJ marker staining among the groups, correlations between intubating ROC dose and NMJ dysmorphism were only found in controls where AChE mismatches with nAChR (EOR) were significant (Figure 5A). We found that among CP subjects, independent of anticonvulsant therapy required elevated ROC doses, there was an increased area of nAChR, and that the distribution of nAChR in patients with CP, depending on the subgroup, exhibited either a positive or a negative correlation with intubating ROC dose (positive for subjects with CP on CAT and negative for subjects with CP not on CAT; Supplemental Table S3). This complex landscape of NMJ morphologies may suggest that increased use of ROC in CP may be due to aspects NMJ morphology alteration but that we may need to consider other components than the AChR receptor target of ROC. These data also indicate that altered distribution of synaptic components are may be associated with elevated ROC, and although ECM expansion was not assessed directly in the current study, it appeared that all participants with CP had elevated Lβ2 in the area of the NMJ (values that were found to correlate with intubating ROC dose; Supplemental Table S4). These differences in the quantity and distribution of AChE and Lβ2 in CP imply a role for altered NMJ microanatomy, which we have seen in other studies [37,43,44,71], and which may be indicative of NMJ dysfunction [72]. Overall, our data are suggestive that NMJ conformation may be related to the amount of ROC needed during surgery, but the pairwise NMJ measures assessed here may not be the best indicators for functional dysmorphism among participants with CP.

The findings of our current study are compelling but necessarily limited by the data available in participant medical records, which made some comparisons challenging. Another potential limitation exists in our reliance on patients presenting for surgery who received ROC, which may represent a sub-population of all individuals with spastic CP, and our reliance on recorded ROC utilization, which is an accurate number that may have been influenced by subjective or complex considerations associated with individual cases. The interpretation of results in this paper is necessarily bounded by these limitations, and extrapolation to the broader CP community may require additional research.

## 5. Conclusions

Overall, our findings provide insight into ROC usage in patients with CP and highlight manifestations of spastic CP. The detailed mechanisms accounting for ROC resistance in children with CP requires further investigation, but a set of complex individual traits may be related to ROC resistance, and ROC resistance may typify a set of broader issues impacting personalized treatment plans for children with spastic CP and considerations around variable drug responses associated with medications targeting NMJs or peripheral neuromotor function. In particular, clinicians should be aware of the possibility that individuals with CP have varied resistance to ROC that may be disease severity and disease etiology dependent with possible relationships to NMJ microstructure.

## Figures and Tables

**Table 1:** Descriptive statistics for Study participants *.

	Control		GMFCS I		GMFCS II		GMFCS III		GMFCS IV		GMFCS V		Total CP	
No. of Patients	73		3		6		6		14		35		64	
No. per Gender	M:31	F:42	M:2	F:1	M:2	F:4	M:1	F:5	M:9	F:5	M:24	F:11	M:38	F:26
Mean Age **	14.9 ± 2.55		11.3 ± 0.58		12.7 ± 3.50		13.7 ± 2.80		11.4 ± 4.07		13.1 ± 4.19		12.7 ± 3.90	
Mean BMI **	23.4 ± 6.43		15.6 ± 1.89		19.7 ± 4.76		21.3 ± 8.67		17.1 ± 3.57		18.4 ± 335		18.0 ± 4.78	
Mean Surgery Duration (hrs) **	7.42 ± 2.19		4.8 ± 1.04		5.40 ± 1.71		5.38 ± 1.73		7.50 ± 3.27		6.67 ± 2.54		6.58 ± 2.61	
Patients on CAT	0		1		2		0		7		23		33	
Patients not on CAT	0		2		4		6		7		12		31	
Hypoxia	0		0		1		3		2		10		16	
IVH/Hemorrhage	0		0		0		0		1		1		2	
HIE	0		0		0		0		3		6		9	
Perinatal Infection	0		0		0		0		1		1		2	
Encephalitis	0		0		0		1		0		1		2	
Peri/Postnatal Trauma	0		0		0		1		1		2		4	
Combination of Above	0		2		2		1		2		4		11	
Other or Not Recorded	0		1		3		0		4		10		18	
Preterm (GA < 37 weeks) ***	5		3		2		5		12		15		37	
Full Term (GA ≥ 37 weeks)	52		0		4		1		2		18		25	
GA Not Recorded	16		0		0		0		0		2		2	

* Data from the 137 included in the study analysis. ** Significant difference between diagnostic groups for mean age, BMI, and length of surgery (*p* = 5.0 × 10^−5^, 4.3 × 10^−9^, and 0.04 respectively, MWU). *** Average preterm GA (weeks): CP = 29.8 ± 4.3, Control = 33.5 ± 4.4. Abbreviations: GMFCS = Gross Motor Function Classification System; BMI = Body Mass Index; CAT = Chronic Anticonvulsant Therapy; IVH = Intraventricular Hemorrhage; HIE = Hypoxic Ischemic Encephalopathy; GA = Gestational Age.

**Table 2:** Correlation of rocuronium with birth statistics and serum analytes.

Intubating Rocuronium Dose													
Measure	Reference Range	Control				Cerebral Palsy Not on CAT				Cerebral Palsy on CAT			
		*n*	Mean ± SEM	Pearson r	*p* Value	*n*	Mean ± SEM	Pearson r	*p* Value	*n*	Mean ± SEM	Pearson r	*p* Value
BW (kg) ^1^	2.5–4.0	44	3.53 ± 0.10	−0.166	0.281	24	1.58 ± 0.18	0.544	0.006 **	29	2.58 ± 0.22	0.160	0.408
GA (weeks) ^1^	37–40	58	39.3 ± 0.31	−0.142	0.289	30	32.0 ± 0.90	0.416	0.022 *	32	35.3 ± 1.13	0.247	0.173
BW-by-GA	0.07–0.10	42	0.09 ± 0.00	−0.148	0.35	24	0.05 ± 0.00	0.510	0.011 *	29	0.07 ± 0.00	0.117	0.545
Serum ALT ^2^	0–35 U/L	0	NA	NA	NA	14	25.4 ± 2.10	0.286	0.322	29	26.8 ± 2.49	0.283	0.136
Serum Bilirubin ^2^	0.3–1.2 mg/dL	0	NA	NA	NA	14	0.54 ± 0.08	−0.062	0.833	29	0.27 ± 0.03	−0.153	0.427
Serum Mg ^2^	1.5–2.4 mg/dL	19	2.92 ± 0.19	0.255	0.291	4	3.27 ± 0.25	0.117	0.883	13	3.23 ± 0.30	−0.219	0.473
Serum Tri ^2^	<250 mg/dL	19	56.9 ± 11.1	−0.042	0.864	4	19.6 ± 10.3	−0.768	0.232	13	24.6 ± 6.60	0.032	0.918
**Total Rocuronium Dose**													
**Measure**	**Reference Range**	**Control**				**Cerebral Palsy Not on CAT**				**Cerebral Palsy on CAT**			
		***n***	**Mean ± SEM**	**Pearson r**	***p* Value**	***n***	**Mean ± SEM**	**Pearson r**	***p* Value**	***n***	**Mean ± SEM**	**Pearson r**	***p* Value**
BW (kg) ^1^	2.5–4.0	44	3.53 ± 0.10	−0.166	0.281	24	1.58 ± 0.18	0.581	0.003 **	29	2.58 ± 0.22	0.160	0.408
GA (weeks) ^1^	37–40	58	39.3 ± 0.31	−0.142	0.289	30	32.0 ± 0.90	0.432	0.017 *	32	35.3 ± 1.13	0.247	0.173
BW-by-GA	0.07–0.10	42	0.09 ± 0.00	−0.148	0.35	24	0.05 ± 0.00	0.555	0.005 **	29	0.07 ± 0.00	0.117	0.545
Serum ALT ^2^	0–35 U/L	0	NA	NA	NA	14	25.4 ± 2.10	0.287	0.320	29	26.8 ± 2.49	0.454	0.013 *
Serum Bilirubin ^2^	0.3–1.2 mg/dL	0	NA	NA	NA	14	0.54 ± 0.08	−0.154	0.600	29	0.27 ± 0.03	0.040	0.837
Serum Mg ^2^	1.5–2.4 mg/dL	19	2.92 ± 0.19	0.259	0.285	4	3.27 ± 0.25	−0.573	0.427	13	3.23 ± 0.30	−0.051	0.869
Serum Tri ^2^	<250 mg/dL	19	56.9 ± 11.1	0.016	0.948	4	19.6 ± 10.3	−0.260	0.740	13	24.6 ± 6.60	−0.324	0.279

^1^ Nemours Kids Health, ^2^ Merck Manual; Pearson r statistic, *p* < 0.05 = *, *p* < 0.01 = **. Abbreviations: CAT = Chronic Anticonvulsant Therapy; SEM = Standard Error of Measurement; BW = Birth Weight; GA = Gestational Age; ALT = Alanine Transaminase; Mg = Magnesium; Tri = Triglycerides.

## Data Availability

The data presented in this study are available from the corresponding author on reasonable request. The primary data are not publicly available to protect the privacy of research participants.

## Supplementary Materials

The following are available online at https://www.mdpi.com/article/10.3390/jpm11080765/s1, Figure S1: Sensitivity Analysis of Rocuronium Dosage by Anesthesiologist, Figure S2: Correlation of total rocuronium dose with birth statistics and serum analytes, Figure S3: Non-Significant findings for NMJ measures vs. intubating ROC dose, Table S1: Rocuronium utilization by category and anticonvulsant type, Table S2: Mean Intubating and Total ROC dose by Etiology, Table S3: Correlation of Rocuronium use and NMJ dysmorphism scores by group, Table S4: Correlation of Rocuronium use and NMJ dysmorphism scores by diagnosis, Video S1: Comparison of control and CP NMJ in 3D.

## Abbreviations

ACL	Anterior cruciate ligament
AChE	Acetylcholinesterase
ALT	Alanine transaminase
ANOVA	Analysis of variance
BMI	Body mass index
BW	Birth weight
CAT	Chronic anticonvulsant therapy
CP	Cerebral palsy
CYP 3A4	Cytochrome P450 3A4
EMR	Electronic Medical Record
FET	Fisher’s exact test
FT	Full-term
GA	Gestational age
GMFCS	Gross motor function classification system
HIE	Hypoxic ischemic encephalopathy
IgG	Immunoglobulin G
IRB	Institutional review board
IVH	Intraventricular hemorrhage
KW	Kruskal Wallis
Lb2	Laminin beta 2
LBW	Low birth weight
Mg	Magnesium
MWU	Mann Whitney U
nAChR	Nicotinic acetylcholine receptor
NBW	Normal birth weight
NMBA	Neuromuscular blocking agent
NMJ	Neuromuscular junction
PT	Pre-term
ROC	Rocuronium bromide
SEM	Standard error of the mean
Tri	Triglycerides
TWA	Two way ANOVA
