# Supplementary material for: Resistance to Neuromuscular Blockade by Rocuronium in Surgical Patients with Spastic Cerebral Palsy
Source: J Pers Med. 2021 Aug 3;11(8):765. doi: 10.3390/jpm11080765 (PMC8400439; doi:10.3390/jpm11080765)
Supplement: Supplementary file 1 [file jpm-11-00765-s001.zip › jpm-1265380-supplementary/jpm-1265380-supplementary.pdf]

SUPPLEMENTAL INFORMATION

Resistance to neuromuscular blockade by rocuronium in surgical patients with spastic cerebral palsy.

Stephanie Lee, Karyn Robinson, Madison Lodge, Mary Theroux, Freeman Miller, and Robert Akins Jr.

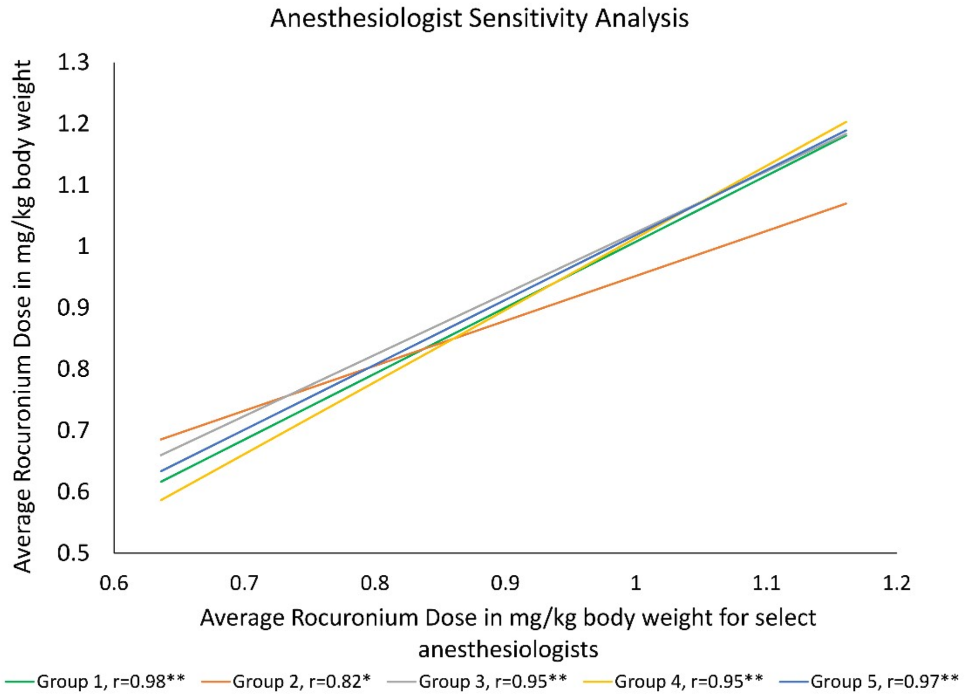

Supplemental Figure 1: Sensitivity Analysis of Rocuronium Dosage by Anesthesiologist. A sensitivity analysis was prepared such that mean ROC doses by GMFCS score were correlated against the same means but with the data of 5 anesthesiologists was removed. Pearson r values are shown in the legend with significance values represented by a number of asterisks where * = $p<0.05$, **=$p<0.01$. Results show that effects seen in ROC dosing were not attributed to a specific anesthesiologist.

A)

Group	Control				Cerebral Palsy			
Measure	N	Mean ± SEM	Median	Range	N	Mean ± SEM	Median	Range
Total	73	0.64 ± 0.03	0.59	1.04	64	0.99 ± 0.08	0.86	4.11
F-Term	52	0.64 ± 0.03	0.6	0.8	25	1.29 ± 0.16	1.14	3.88
P-Term	6	0.65 ± 0.09	0.55	0.55	37	0.76 ± 0.06	0.71	1.28
LBW	2	0.89 ± 0.13	0.89	0.26	29	0.76 ± 0.06	0.81	1.17
NBW	42	0.63 ± 0.03	0.6	0.77	24	1.27 ± 0.17	1.14	3.92
Male	31	0.66 ± 0.04	0.61	1.04	38	1.03 ± 0.12	0.92	4.11
Female	42	0.61 ± 0.04	0.55	0.81	26	0.93 ± 0.08	0.85	1.97
GMFCS I	- -	- -	NA	NA	3	0.64± 0.03	0.62	0.11
GMFCS II	- -	- -	NA	NA	6	0.75 ± 0.20	0.67	1.41
GMFCS III	- -	- -	NA	NA	6	0.82 ± 0.11	0.83	0.76
GMFCS IV	- -	- -	NA	NA	14	0.80 ± 0.12	0.72	1.31
GMFCS V	- -	- -	NA	NA	35	1.16 ± 0.12	1.1	4.02
Unknowns: Control, Term n = 15, BW n = 29; Cerebral Palsy, Term n= 2, BW n = 11								

B)

Group	Cerebral Palsy (CAT group)				Cerebral Palsy (Non-CAT group)			
Measure	N	Mean ± SEM	Median	Range	N	Mean ± SEM	Median	Range
Total	33	1.15 ± 0.13	1	3.99	31	0.81 ± 0.07	0.81	2.07
F-Term	20	1.32 ± 0.19	1.16	3.88	5	1.17 ± 0.29	0.87	1.66
P-Term	12	0.83 ± 0.11	0.78	1.16	25	0.73 ± 0.06	0.71	1.09
LBW	9	0.84 ± 0.11	0.85	1.05	20	0.73 ± 0.08	0.76	1.09
NBW	20	1.27 ± 0.19	1.14	3.92	4	1.26 ± 0.36	1.02	1.57
Male	21	1.26 ± 0.19	1.14	3.99	14	0.74 ± 0.08	0.72	1.09
Female	12	0.96 ± 0.09	0.91	1.15	17	0.90 ± 0.13	0.84	1.97
GMFCS I	1	0.71	0.71	0	2	0.61 ± 0.01	0.61	0.02
GMFCS II	2	1.05 ± 0.58	1.05	1.15	4	0.59 ± 0.14	0.67	0.63
GMFCS III	- -	- -	NA	NA	6	0.82 ± 0.11	0.83	0.76
GMFCS IV	7	1.24 ± 0.17	0.93	1.22	7	0.66 ± 0.13	0.57	0.86
GMFCS V	23	1.15 ± 0.13	1	3.99	12	1.00 ± 0.15	0.81	2.07
Unknowns: CP-CAT Term n = 1, BW n = 4; CP-Non-CAT, Term n = 1, BW n = 7.								

C)

Rocuronium dose (mg/kg body weight) by Anticonvulsant Hepatotoxicity Rating

Drug Brand Name(s)	Rating*	N	Intubating Dose†			Total Dose‡		
			Mean ± SEM	Median	Range	Mean ± SEM	Median	Range
Luminal, Tegretol, Valproate, Lamictal	A	17	1.22 ± 0.22	0.93	3.88	2.31 ± 0.55	1.4	8.82
Keppra, Topomax	C	14	1.10 ± 0.12	1.2	1.33	1.97 ± 0.24	2.05	2.68
Klonopin, Trileptal	D	2	1.43 ± 0.19	1.43	0.38	2.58 ± 0.26	2.58	1.43

* Hepatotoxicity rating based on LiverTox: Clinical and Research Information on Drug-Induced Liver Injury. Bethesda (MD): National Institute of Diabetes and Digestive and Kidney Diseases; 2012-. Anticonvulsants. [Updated 2019 Apr 18]. Available from: https://www.ncbi.nlm.nih.gov/books/NBK548365/

† Intubating doses were not significantly different, P = 0.550, Kruskal-Wallis

‡ Total doses were not significantly different, P = 0.550, Kruskal-Wallis, P = 0.686, Kruskal-Wallis

Supplemental Table 1: Rocuronium utilization by category and anticonvulsant type. A-B: Tables of intubating ROC dose in mg/kg body weight, for all groups shown in Figure 1, first categorized by diagnosis (Table A) and then by patients with CP who were on vs not on chronic anticonvulsant therapy (Table B). Rocuronium dose was not significantly different ($p = 0.718$, KW) between patients with CP when categorized by the likelihood score of the anticonvulsant used (Table C, A = higher hepatotoxicity, D = lower hepatotoxicity).

Rocuronium dose by Etiology of Cerebral Palsy			
**Etiology**	**N**	**Mean Intubating Roc Dose**	**Mean Total ROC Dose**
Hypoxia	16	0.89 ± 0.09	1.59 ± 0.22
IVH/Hemorrhage	2	0.87 ± 0.29	1.21 ± 0.64
HIE	9	1.07 ± 0.22	1.71 ± 0.37
Perinatal Infection	2	1.86 ± 0.24	3.32 ± 1.70
Encephalitis	2	1.06 ± 0.60	1.48 ± 1.01
Peri/Postnatal Trauma	4	1.64 ± 0.91	3.60 ± 1.96
Combination	11	0.78 ± 0.10	1.05 ± 0.20
Other/Not Noted	18	0.91 ± 0.09	1.46 ± 0.25
Intubating Dose, P = 0.600; Total Dose, P = 0.515, KW			

Supplemental Table 2: Mean Intubating and Total ROC dose by Etiology. Mean intubating and total rocuronium dose was compared when patients with CP were grouped by suspected disease etiology. For patients in this study, results showed that increased need for rocuronium was not etiology dependent.

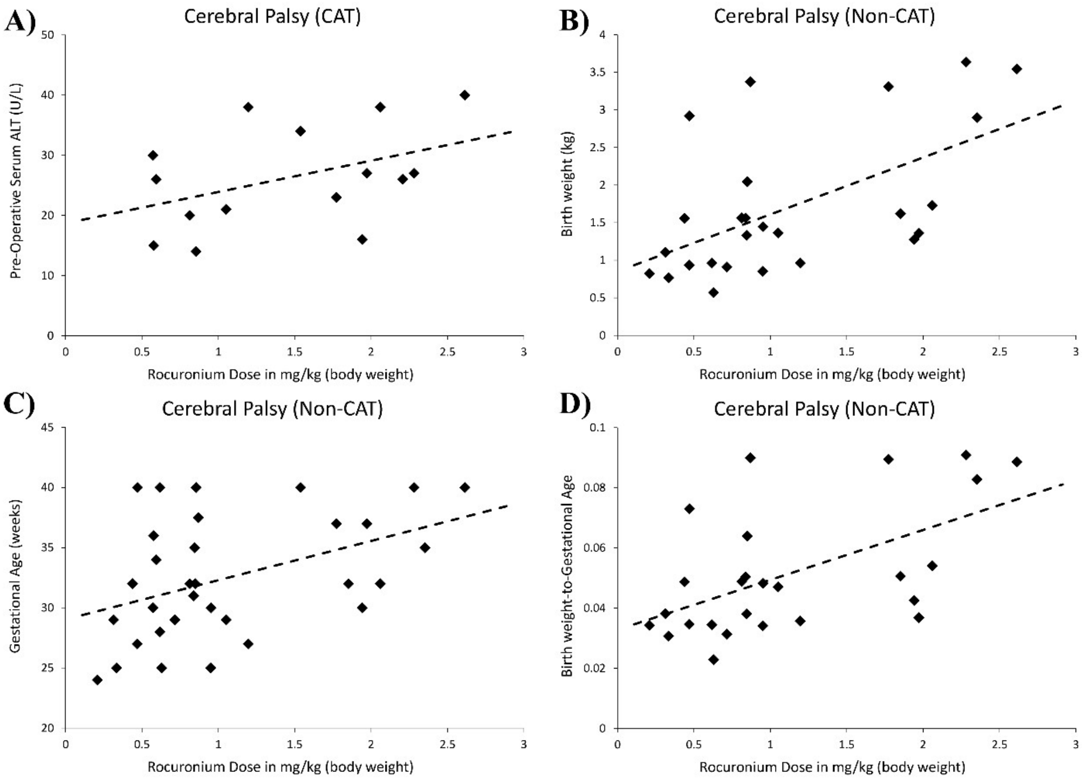

Supplemental Figure 2: Correlation of total rocuronium dose with birth statistics and serum analytes. Graphical representation of significant findings represented in Table 2. Participants not on CAT exhibited significant correlations between total ROC dose and birthweight (A, Pearson r = 0.581, p=0.003), gestational age (B, Pearson r = 0.432, p=0.017), and the ratio of birthweight to gestational age (C, Pearson r = 0.555, p=0.005). For total ROC dose only CP participants receiving CAT exhibited a significant correlation between ROC dose and pre-operative ALT levels (D, Pearson r = 0.454, p=0.013).

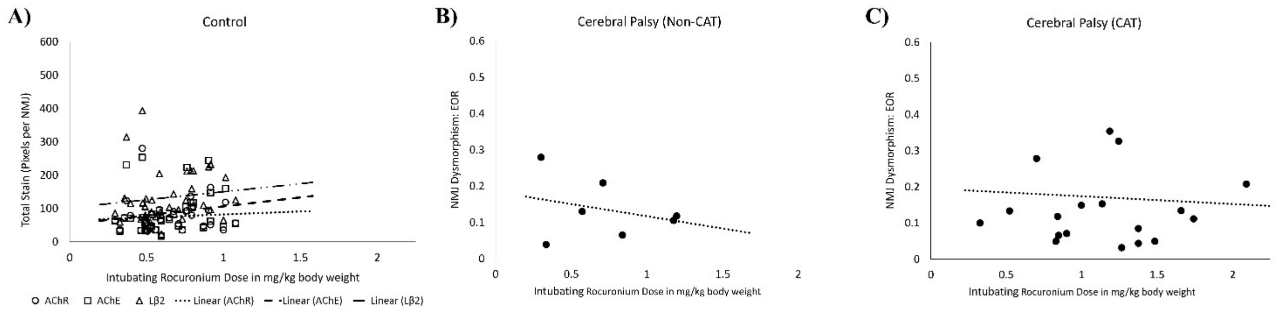

Supplemental Figure 3: Non-Significant findings for NMJ measures vs intubating ROC dose. For controls total distribution of each NMJ component normalized to body weight, (AChR, AChE, and LB2), did not correlate with intubating ROC dose (**A**). Unlike their control counterparts, patients with CP in both sub-groups did not exhibit a correlation between intubating ROC dose and the NMJ dysmorphism finding of excess AChE staining outside of the area colocalized with AChR (**B** and **C**).

Correlation analysis for *intubating* rocuronium dose and NMJ organization score

Measure	Control		Cerebral Palsy not on CAT		Cerebral Palsy on CAT	
	Pearson r	Statistic	Pearson r	Statistic	Pearson r	Statistic
**EOL**	0.265	0.059	-0.425	0.171	-0.253	0.141
**LOE**	0.152	0.188	0.103	0.413	-0.041	0.431
**ROL**	-0.195	0.127	0.155	0.370	-0.262	0.133
**LOR**	0.237	0.082	0.066	0.444	-0.072	0.382
**ROE**	0.180	0.147	-0.437	0.163	-0.145	0.271
**EOR**	**.338***	**0.022**	-0.295	0.260	-0.108	0.326
**Total E**	0.177	0.150	**-.851****	**0.008**	0.226	0.169
**Total L**	0.129	0.226	**-.908****	**0.002**	**.428***	**0.030**
**Total R**	0.077	0.328	**-.840****	**0.009**	**.394***	**0.043**

Correlation analysis for *total* rocuronium dose and NMJ organization score

Measure	Control		Cerebral Palsy not on CAT		Cerebral Palsy on CAT	
	Pearson r	Statistic	Pearson r	Statistic	Pearson r	Statistic
**EOL**	0.225	0.093	-0.187	0.344	-0.372	0.053
**LOE**	0.122	0.239	-0.060	0.449	-0.046	0.424
**ROL**	-0.185	0.140	-0.034	0.471	**-.383***	**0.048**
**LOR**	0.166	0.167	0.140	0.382	-0.120	0.307
**ROE**	0.143	0.203	-0.334	0.232	0.001	0.498
**EOR**	**.333***	**0.023**	0.281	0.271	-0.220	0.175
**Total E**	0.203	0.118	-0.591	0.081	0.271	0.124
**Total L**	0.162	0.173	**-.831***	**0.010**	**.557****	**0.005**
**Total R**	0.144	0.201	**-.725***	**0.033**	**.574****	**0.004**

Supplemental Table 3: Correlation of Rocuronium use and NMJ dysmorphism scores by group.

Pearson correlation analysis of NMJ dysmorphism score against both intubating (top) and total (bottom) rocuronium dose was compared among all three cohorts: control, cerebral palsy on CAT, and cerebral palsy not on CAT. Significant findings within the group of CP participants were not the same when the group was split based on anticonvulsant use and were only in the measures correlated against total rocuronium dose.

Correlation analysis for *intubating* rocuronium dose and NMJ organization score

Measure	Control		Cerebral Palsy	
	Pearson r	Statistic	Pearson r	Statistic
**EOL**	0.265	0.059	-0.187	0.175
**LOE**	0.152	0.188	-0.063	0.377
**ROL**	-0.195	0.127	-0.088	0.332
**LOR**	0.237	0.082	-0.097	0.316
**ROE**	0.180	0.147	-0.110	0.292
**EOR**	**.338***	**0.022**	-0.082	0.343
**Total E**	0.177	0.150	-0.005	0.490
**Total L**	0.129	0.226	0.087	0.333
**Total R**	0.077	0.328	0.094	0.320

Correlation analysis for *total* rocuronium dose and NMJ organization score

Measure	Control		Cerebral Palsy	
	Pearson r	Statistic	Pearson r	Statistic
**EOL**	0.225	0.093	-0.273	0.084
**LOE**	0.122	0.239	-0.090	0.328
**ROL**	-0.185	0.140	-0.185	0.177
**LOR**	0.166	0.167	-0.136	0.250
**ROE**	0.143	0.203	0.032	0.436
**EOR**	**.333***	0.023	-0.146	0.234
**Total E**	**0.203**	0.118	0.071	0.362
**Total L**	0.162	0.173	0.198	0.162
**Total R**	0.144	0.201	0.248	0.106

Supplemental Table 4**: **Correlation of Rocuronium use and NMJ dysmorphism scores by diagnosis. Data was pooled for CP cohort to evaluate how NMJ dysmorphism or total stain distribution per NMJ correlate with both intubating and total rocuronium dose. When analyzing the pooled data, there are no longer correlations between any measure and ROC dose for the CP cohort.

Supplemental Videos: Comparison of control and CP NMJ in 3D. Video files attached demonstrate the degree of NMJ disorganization in participants with cerebral palsy. Video files attached correspond to the NMJ images shown in figure 3.
