# Supplementary material for: Resistance to Neuromuscular Blockade by Rocuronium in Surgical Patients with Spastic Cerebral Palsy
Source: J Pers Med. 2021 Aug 3;11(8):765. doi: 10.3390/jpm11080765 (PMC8400439; doi:10.3390/jpm11080765)
Supplement: Supplementary file 1 [file jpm-11-00765-s001.zip › jpm-1265380-supplementary/jpm-1265380-video.pdf]

Supplementary Video Link:

https://drive.google.com/drive/folders/1twnUt1j3gxLkIuY7cOuG1lqUM4xJUoHh?usp=sharing
